# The diagnosis and surgical management of pulmonary sequestration in adults: a case series from a single centre in the UK

**DOI:** 10.1007/s12055-023-01589-2

**Published:** 2023-09-04

**Authors:** Ashar Asif, Daniel Lilley, Sherene Howard-Walker, Shereen Ajab, Syed Suhail Qadri

**Affiliations:** 1 Castle Hill Hospital, Hull University Teaching Hospitals NHS Trust, Castle Rd, Cottingham, HU16 5JQ UK; 2 Hull York Medical School, University Rd, Heslington, York YO10 5DD UK

**Keywords:** Pulmonary sequestration, Robotic lung resection, Video-assisted thoracoscopic surgery, Computed tomography, Magnetic resonance imaging

## Abstract

Pulmonary sequestration (PS) is a rare congenital malformation where extrapulmonary lung tissue receives systemic blood supply from an anomalous branch directly from the thoracic or abdominal aorta. Whilst non-malignant, it can often present with similar symptoms as lung cancer. We present a retrospective review of 8 consecutive adult patients undergoing surgical management for PS within a single centre in the UK. Of our cohort, 62.5% had never smoked. PS in the right lung was seen in 62.5% of cases. Anomalous branches of the pulmonary artery, pulmonary vein or coeliac axis supplied 37.5% of the PS seen in our cohort, and 12.5% did not have a radiologically identifiable blood supply. Techniques varied from thoracotomy (*n* = 4), video-assisted thoracoscopic surgery (VATS) (*n* = 3) to robotic resection (*n* = 1) with no intra-operative or post-operative complications reported within hospital. The mean length of stay was 2 days. The post-operative mortality rate was 12.5%; one patient had died following the robotic resection of the mass of pneumonia in the local district hospital 26 days post-operatively after being discharged. No other complications nor recurrence was recorded over the follow-up period. Where pulmonary masses receive blood supply from anomalous branches of the pulmonary vein and coeliac axis, diagnoses of PS should be considered. The clinical feasibility of discharge in 2 days with no symptom recurrence should undergo further investigation with a larger sample size.

## Introduction

Pulmonary sequestration (PS) is a rare congenital malformation where extrapulmonary lung tissue, not communicating with the tracheobronchial tree, receives systemic blood supply from the thoracic or abdominal aorta. PS comprises 0.15–6.4% of all congenital lung lesions [1]. PS can be divided into intralobar and extralobar sequestrations (ILS and ELS); ILSs share the main visceral pleura with the original lung parenchyma whilst ELSs are separated from the lungs by their own pleural lining.

PS can present as recurrent pneumonia, haemoptysis, persistent cough, dyspnoea or incidentally on computed tomography (CT) [2–4]. If symptomatic, excision of the lesion is favoured and has mostly been performed through a thoracotomy or video-assisted thoracoscopic surgery (VATS). However, cases of robotic-assisted resection have also been reported [5, 6].

## Methods

We present a retrospective review of consecutive patients aged over 18 years who were treated surgically for PS between 2017 and 2022 at a single centre. The operative approaches were via thoracotomy, VATS and robotic-assisted resection. We present the pre-operative radiological findings, histological findings, operative approach and post-operative outcomes.

## Results

### Patient demographics

A total of 8 patients were treated for PS. The most common causes for referral were incidental findings on radiographical imaging in asymptomatic patients and haemoptysis. The patient demographics are summarised in Table 1.

**Table 1:** Patient demographics

	Number of cases	Percentage of total cases (%)
Gender		
Men	5	62.5
Women	3	37.5
Age* (years)	26–91	48.9 (mean)
Smokers	3	37.5
Symptoms		
Incidental	3	37.5
Haemoptysis	3	37.5
Recurrent pneumothorax	1	12.5
Recurrent pneumonia	1	12.5

*Age is presented as range and mean

### Radiological findings

All patients underwent pre-operative contrast-enhanced CT imaging; 3 (37.5% of total patients) underwent positron emission tomography (PET) imaging and 1 (12.5%) underwent magnetic resonance imaging (MRI). PET imaging was used by the referring teams as part of further investigation of a radiologically indeterminate mass to assess for potential malignancy. The PET images ranged showing mild to avid fluorodeoxyglucose uptake and activity. The MRI had shown a homogenous, non-enhancing and well-defined lesion with no imaging evidence of feeding aortic vessel. This patient was found to have accessory arterial supply via the inferior pulmonary ligament intraoperatively. All patients had associated significant mediastinal lymphadenopathy. The CT findings are summarised in Table 2.

**Table 2:** Radiological findings

	Number of cases	Percentage of total cases (%)
Classification of pulmonary sequestration		
Intralobar	6	75.0
Extralobar	2	25.0
Localisation of pulmonary sequestration		
Right lung	5	62.5
Lower lobe	3	37.5
Middle lobe	1	12.5
Multiple lobes	1^†^	12.5
Left lung	3	37.5
Lower lobe	3	37.5
Vascular supply		
Intralobar		
Descending thoracic aorta	3	50.0
Pulmonary vein	1	12.5
Coeliac trunk	1	12.5
Multiple	1*	12.5
Unable to identify from imaging	1	12.5
Extralobar		
Descending thoracic aorta	2	25

^†^Pulmonary sequestration was adjacent to both lower and middle lobes

*Received blood supply to their pulmonary sequestration from both the thoracic aorta and pulmonary circulation

**Fig. 1:**
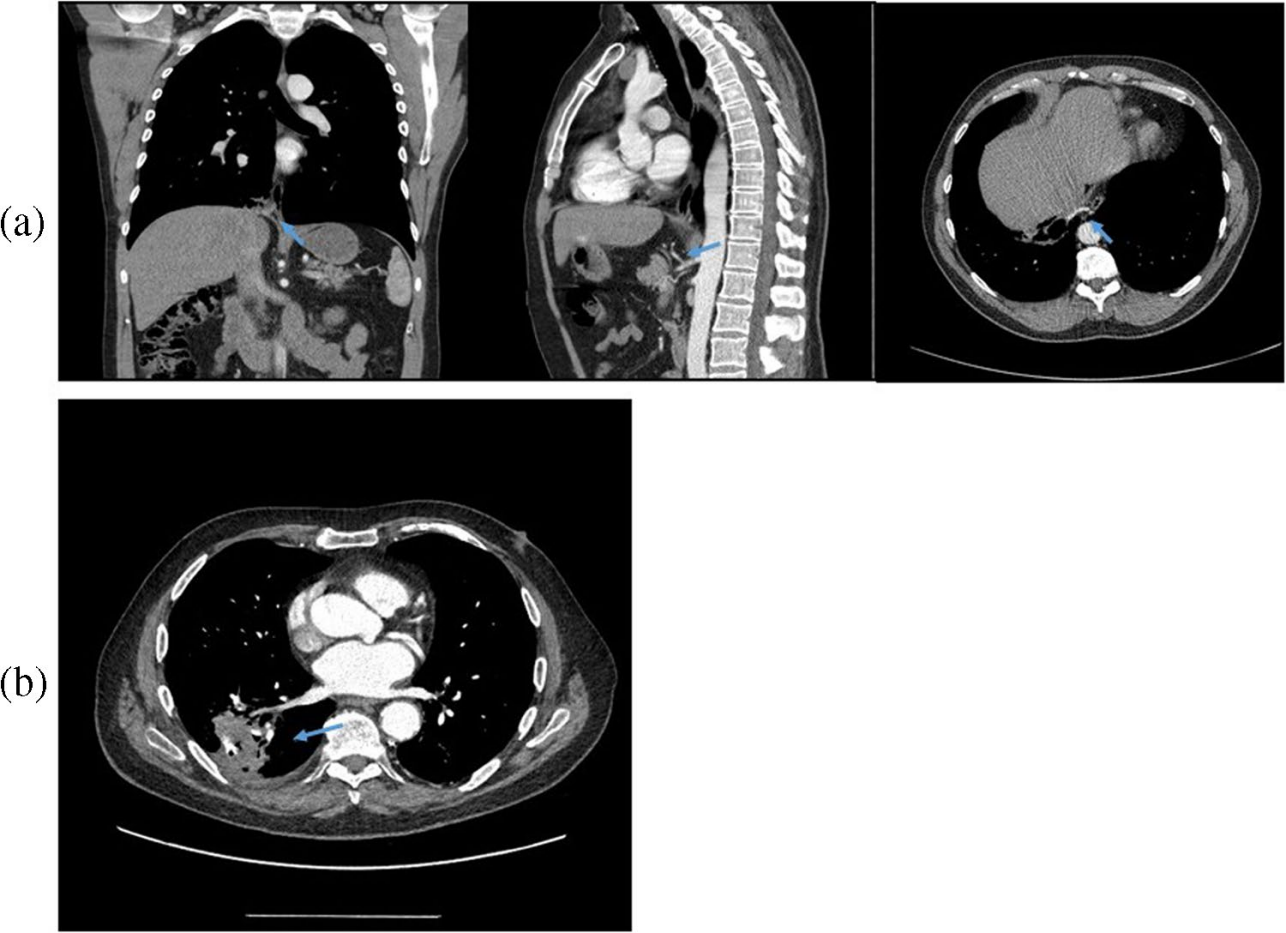
**a** A series of coronal, sagittal and axial contrast-enhanced computed tomography images of the thorax and abdomen identifying branches from the coeliac axis entering through the oesophageal hiatus to supply a small mass of the right lower lobe. **b** Contrast-enhanced CT image of the thorax showing a right lower lobe mass receiving blood supply from the pulmonary vein

Figure 1 demonstrates PS receiving blood supply from aberrant branches of vessels such as the pulmonary vein and coeliac axis, which are not typically described in PS.

### Surgical approach and operative findings

Half of our patient cohort (*n* = 4) underwent a thoracotomy in the lateral decubitus position. The remainder underwent VATS (*n* = 3) and robotic resection (*n* = 1). Robotic and VATS were performed with routine 3-port approach in the lateral decubitus position. The patients with ELS either underwent wedge resection or segmentectomy. The operative approach for intralobar PS was either robotic or VATS lobectomy. For the patient who underwent robotic resection of their intralobar PS, a standard 3-port robotic approach was used. The inferior pulmonary ligament was divided and inferior pulmonary vein was first dissected and isolated before being stapled and divided. The arterial branches to the lower lobe were dissected and isolated before being stapled and divided. The lower lobe bronchus and fissure were also stapled.

**Fig. 2:**
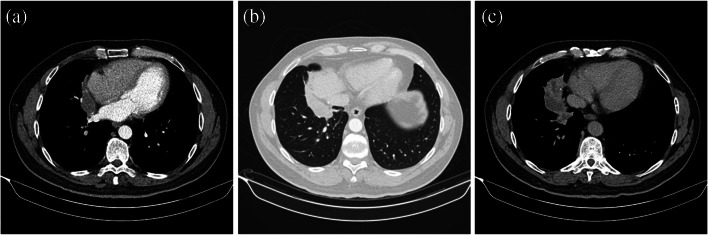
Computed tomography images of an intralobar PS adhered to the (**a**) pericardium and (**b**) diaphragm. **c** The computed tomography shows the mass to be of varying densities

One intralobar PS that was approached by open thoracotomy had multiple aberrant tortuous systemic and pulmonary blood supplies. This approach was taken as the mass adhered to the diaphragm, pericardium, right hilum and lower and middle lobe with significant mediastinal lymphadenopathy (Fig. 2). Here, a thoracoscopic approach may have proven difficult. The sequestration was carefully dissected off the adjacent structures, and veins to the middle and lower lobe were dissected and divided with a vascular stapler. This mass was grossly found to be of mixed firm and cystic consistency.

Tissue and adjacent lymph nodes were sent for histological analysis which showed inflammatory, fibrotic changes with immune cell infiltrates. One mass reached 52 g in weight and was 9 × 7 × 3 cm in size. There was no histological evidence of malignancy in the samples taken from any patient.

### Post-operative outcomes

Average length of stay of 2 days (range 1–3 days), with no in-hospital mortality or morbidity was seen. On discharge, all patients underwent routine post-operative follow-up imaging and review by their operating surgeon within 4 weeks. Following this appointment, patients were either discharged, if all checks were satisfactory, or kept under surveillance of surgical team, if symptoms persisted or there was evidence of disease recurrence. Post-operative complication was seen in one patient, who died post-discharge, on post-operative day 26, after being admitted to his local district general hospital for pneumonia. For the remaining 7 patients, none had reported any symptoms to their respective surgeon, and all follow-up chest X-rays were satisfactory. According to patients’ central records, none had presented to their primary care physician requiring further medical support or re-referral to our team. One patient had a CT 1 year post-operatively with haemoptysis, which was deemed to be secondary to chronic cough. There was no radiological evidence of PS recurrence and no evident cause for the haemoptysis. One patient was experiencing neuropathic thoracic pain near the thoracotomy scar and was started on a course of gabapentin. No patient had been re-referred to our service due to recurrence or worsening of symptoms, or complications over 5 years of follow-up.

## Discussion

PS is a rare malformation, most commonly presenting as an ILS, with quoted rates between 75 and 93% [1, 5–8], whereas we observed 75% of cases being intralobar. We saw the majority of sequestrations in the lower lobes, with equal distribution between left and right lower lobes. Other published studies quote approximately 66–77% of PS cases being located in the left lower lobe [5, 7, 8].

We also observed that sequestrations receiving blood supply from branches other than from the aorta directly, as classically described, tended to be seen in intralobar PS. Where pulmonary masses receive blood supply from a single or multiple anomalous branches, other than the thoracic aorta as expected, diagnoses of PS should be considered. We observed 2 patients with sequestrations receiving blood supply from the pulmonary circulation, one from an anomalous branch of the coeliac axis and another from an accessory artery within the inferior pulmonary ligament, and all samples histologically confirmed PS. Similarly, other recent case series have presented PS receiving blood supply from the coeliac trunk, subclavian artery or pulmonary artery [9, 10]. Given the increasing frequency of varying, and in some cases multiple, blood supply, perhaps, the definition of PS receiving blood supply from an aberrant branch of the aorta [11] may need reconsidering.

The average length of stay in our cohort was 2 days with no concerns on follow-up. In other case series, the mean length of stay has been quoted as between 4 and 6 days [5, 9, 10]. This could be explained with our patient cohort having a younger mean age and with less comorbidities, therefore allowing for a swift recovery. We have demonstrated that it is feasible to have shorter length of stay following PS resection, thereby reducing length of stay for patients and the associated costs and risks of hospital-acquired infection.

Within our single centre, we have observed good outcomes from either thoracotomy or VATS resection of the lesion. Good outcomes can be obtained despite advanced age, even up to 91 years in our series. However, as a single centre retrospectively reviewing a small cohort, there are limitations. As a rare condition, a single centre may not be presented with many patients with PS. The findings we do present, however, do demonstrate safe outcomes and feasibility for further adequately powered prospective studies on a multicentre level for the operative management of patients with PS.

Generally, the small incisions associated with VATS and robotic resection have the benefit of reduced infection rates and faster post-operative recovery. We have observed good post-operative outcomes with VATS resection and therefore have demonstrated feasibility of using minimally invasive surgery on PS, affirming observed findings published elsewhere [5, 6]. However, our patient who underwent robotic resection of their PS had died, which is contrary to what is published [12]. Given the paucity of evidence for the robotic resection, we suggest further investigation with larger focussed studies into its use in this context.

## Data Availability

Data is available upon request to the corresponding author.
